# Improving the Dimensional Stability and Mechanical Properties of AISI 316L + B Sinters by Si_3_N_4_ Addition

**DOI:** 10.3390/ma12111798

**Published:** 2019-06-03

**Authors:** Mateusz Skałoń, Ricardo Buzolin, Jan Kazior, Christof Sommitsch, Marek Hebda

**Affiliations:** 1 IMAT Institute of Materials Science, Joining and Forming, Graz University of Technology, Kopernikusgasse 24/1, 8010 Graz, Austria; mateusz.skalon@tugraz.at (M.S.); ricardo.buzolin@tugraz.at (R.B.); christof.sommitsch@tugraz.at (C.S.); 2 Institute of Materials Engineering, Cracow University of Technology, Cracow, 24 Warszawska ave, 31-155 Kraków, Poland; kazior@mech.pk.edu.pl

**Keywords:** boron, 316L, silicon nitride, shape distortion, liquid phase sintering, mechanical properties

## Abstract

The following paper describes a new and effective method to obtain high-density sinters with simultaneously decreased distortions, produced by one press and sinter operation. This effect was achieved through the induced disappearance of the eutectic liquid phase. The study was carried out on AISI 316L stainless steel powder that was mixed with elemental boron and silicon nitride. Boron was used as a sintering process activator. The scientific novelty of this publication consists of the use of a silicon nitride as a solid-state nitrogen carrier that was intended to change the borides’ morphology by binding boron. Based on the thermodynamic calculations, 20 blends of various compositions were tested for physical properties, porosity, microstructure, and mechanical properties. Moreover, phase compositions for selected samples were analyzed. It was shown that the addition of silicon nitride as a nitrogen carrier decreases the boron-based eutectic phase volume and both increases the mechanical properties and decreases after-sintering distortions. An explanation of the observed phenomena was also proposed.

## 1. Introduction

The addition of boron to ferrous alloys has been of interest to numerous researchers [1,2,3,4,5,6]. The main cause of this is the eutectic reaction between iron and boron, resulting in the creation of a liquid phase that efficiently intensifies the sintering process by enhancing the diffusion [4,5]. Even small amounts of boron (0.4–0.6 wt%) added to ferrous powder may result in great sinter densification, reaching almost full relative density [5]. Although the influence of boron addition has already been well elaborated, its application is still limited due to high distortions that accompany the densification process induced by a large quantity of the liquid phase [6,7]. According to the literature [8,9,10,11,12], the sintering process is activated through the presence of a liquid phase in three stages: (i) The initial stage is solubility and rearrangement, (ii) the intermediate stage is solution and reprecipitation, (iii) and finally, microstructural coarsening [12]. Unfortunately, boron is almost non-soluble in an iron-based solid matrix, so it remains at the grain boundaries, in the form of hard and brittle borides, after the sintering process, creating an almost continuous network surrounding the grains [1,3,6,13,14,15,16,17]. These borides separate the neighboring grains, significantly influencing the mechanical properties. Controlling these borides by changing their morphology or crystallographic structure would contribute to the development of the microstructures’ optimization process that would allow maximization of the desired properties. It should be noted that nowadays the issues of design and optimization of properties are a widely studied aspect by many authors [18,19,20,21,22,23]. Moreover, it will allow the application field of stainless-steel parts manufactured through powder metallurgy (PM) to be broadened.

Silicon nitride in the presence of iron oxides decomposes, resulting in silicon oxide and free nitrogen after reacting with oxygen [24]. During sintering, materials with a boron addition in a nitrogen atmosphere result in boron nitride due to the high affinity of those two elements [2,6,13]. The objective of this paper is to induce the disappearance of the boron-based eutectic liquid by the addition of silicon nitride as a nitrogen carrier. The investigation was carried out on the commonly investigated AISI 316L powder [14,25,26,27,28,29]. It was expected that by delivering only a small and controlled amount of nitrogen, it would be possible to alter the amount of solidified borides and, therefore, influence the mechanical properties by increasing the contact surface among grains.

## 2. Materials and Methods

Water-atomized austenitic AISI 316L stainless steel, provided by Höganäs AB (Höganäs, Sweden), was used as a base powder. Its chemical composition and grain size distribution are presented in Table 1 and Table 2, respectively.

Elemental, amorphous boron powder, particle size < 1 µm, (Sigma Aldrich, Poznań, Poland) and silicon nitride, particle size < 1 µm, (Sigma Aldrich, Poznań, Poland) were used as additions. Boron was added in an amount ranging up to 0.4 wt% while Si_3_N_4_ was added in an amount calculated in relation to boron to assure a comparable nitrogen to boron molar ratio. Blend compositions are presented in Table 3.

Blends were obtained by 24-hour mixing in a Turbula device. Green compacts were manufactured using a 600 MPa one-sided cold pressing procedure. The die and punch walls were layered with zinc stearate lubricant. Then, the compacts were sintered in a tubular furnace (Nabertherm P330, Merazet, Poznań, Poland) in a 99.9999% purity hydrogen (provided by Air Liquide, Cracow, Poland) atmosphere. The hydrogen flow was set at 6 l*h^−1^. The sintering profile was based on previous investigation [14,16,17]. The sintering temperature profile consisted of heating at a rate of 10 °C/min up to 1240 °C, isothermal sintering for 30 min, and cooling to room temperature at a rate of 20 °C/min. For the sintering operation, samples were placed with the pressing side facing upwards. To investigate the microstructures, Ø20 × 5 mm cylindrical samples were manufactured. The same samples were used to evaluate the density, dimensional tolerances, and microstructural analysis. In total, 30 × 12 × 6 mm prismatic samples were produced for transverse rupture strength (TRS) measurements. Tests were performed using MTS Criterion (model 43) testing equipment with a 0.002 mm*s^−1^constant head movement. The water displacement method (according to the PN-EN ISO 3369:2010 standard) was used to measure the densities of the samples. A weighing scale (Radwag XA/60/220/Y, Radwag, Cracow, Poland) was used (linearity ± 0.05 mg, accuracy: 0.01 mg). The dimensional check after sintering was carried out by measuring the height of the sample using a mechanical height gauge (Cromwell, Leicester, England) equipped with a needle tip of a 0.005 mm accuracy. Porosity analysis was performed by the combined means of optical microscopy (Nikon Eclipse ME 600, Nikon, Warsaw, Poland) and by ImageJ software (imagej.net) (9 pictures for each sample; each picture with a 0.48 mm^2^ area). The average grain size was determined using image analysis. The roundness factor was defined as described in Equation (1) (below):

Round = 4 × π × Area / Perimeter^2^. (1)

Microstructures were revealed by etching using a glycerine based 50% aqua regia at 80 °C for 5 to 10 s. The amount of borides was calculated using image analysis techniques by analyzing an approximately 8 mm^2^ area of pictures taken from near-center spots of the sample cross-section. Thermo-Calc software (v.3.0, Solna, Sweden) with the TCFE6 database was used for thermodynamic calculations. A para-equilibrium state was obtained using the Scheil–Gulliver solidification model with a back-diffusion of boron. Additionally, for the calculations, the full spectrum of phases was taken into consideration [30,31]. Electrochemical tests, like open circuit potential (OCP) variation with time and potentiodynamic polarization, were performed using an Autolab PGSTAT128N potentiostat/galvanostat and NOVA software (Metrohm Autolab B.V., Utrecht, The Netherlands). Measurements were carried out in a 3.5% NaCl solution at ambient temperature according to the methodology described by Szewczyk-Nykiel et al. [32].

## 3. Results and Discussion

### 3.1. Changes in Phase Composition

**Figure 1:**
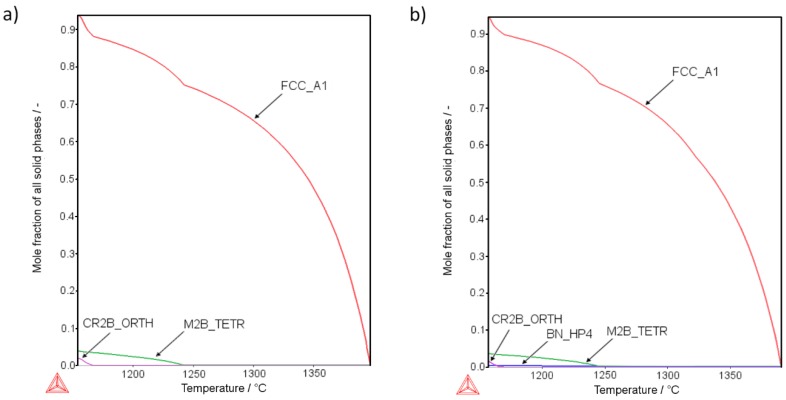
Scheil–Gulliver solidification plots of samples (**a**) 4-0 and (**b**) 4-8. Where: FCC_A1—Austenite, CR2B_ORTH—Orthorhombic (chromium, iron) boride, M2B_TETR—tetragonal (iron, chromium, molybdenum) boride, BN_HP4—Wurtzite-structured boron nitride.

Figure 1 presents the thermodynamic calculations for the AISI 316L phase composition changes along the decreasing temperature curve using the Scheil–Gulliver model with back-diffusion of boron. The calculations show the results for a sample modified with the highest addition of boron (Sample 4-0, Figure 1a) and also for the highest additions of both boron and silicon nitride (Sample 4-8, Figure 1b). The calculations showed that introducing even small amounts of silicon nitride could result in the formation of BN instead of metal borides (Figure 1a,b). According to the calculation results, the more boron was added to the base material, the more borides (in terms of volume) were created. Also, the more silicon nitride was added, the more BN phase appeared, reducing the amount of borides. Detailed numerical and graphical results for all samples are available in the Supplementary Data: Figures S1–S19 and in Table S1.

According to the calculations, the solidification process of the liquid phase in sample 4-0 results in the creation of two types of borides: (i) Cr_2_B with an orthorhombic crystallographic structure, and (ii) M_2_B with a tetragonal crystallographic structure. At room temperature, their molar fraction in the microstructure was equal to 0.0378 (3.78%) and 0.0202 (2.02%), respectively (Figure 1a). In accordance with the simulation, the introduction of both boron and the silicon nitride (sample 4-8) results in the reaction of boron with nitrogen (Figure 1b). Consequently, the refractory boron nitride is created, which is solid at the sintering temperature. As a result of the sintering process, the total volume of borides present at room temperature should be approximately 5.08 vol.% (3.45 vol.% M_2_B-type borides, 1.56 vol % Cr_2_B-type borides). Apart from this, the 0.49 vol.% boron nitride is also expected, which should boost the mechanical properties of a sinter by improving the contact surface among the grains. This calculation remains coherent with findings of Soyama et al. [2], who also found h-BN created in situ.

Detailed calculations show that, at room temperature, two kinds of borides should be present, tetragonal crystallographic M_2_B boride and orthorhombic crystallographic Cr_2_B. Both borides show a similar composition in both samples (4-0 and 4-8) irrespective of the silicon nitride addition (Supplementary Data: Figures S20–S23). Interestingly, calculations indicate that when the Si_3_N_4_ is added, BN is created mostly at the expense of Cr_2_B boride. This effect may lead to a complete disappearance of the Cr_2_B boride, e.g., sample 1-8 (Table S1 in Supplementary Data).

**Figure 2:**
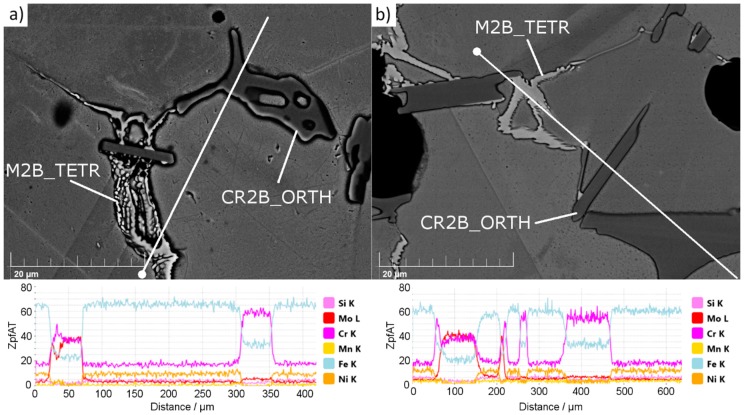
Scanning Electron Microscope Energy-Dispersive X-ray spectroscopy (SEM EDX) linear scans of borides found in (**a**) sample 4-0 and (**b**) sample 4-8. White dots mark the starting points of the scans.

Figure 2 presents the results of an SEM EDX (Scanning Electron Microscope Energy-Dispersive X-ray spectroscopy, MIRA3, TESCAN, Czech Republic) linear scan performed for secondary phases observed in the structure of sample 4-0 (Figure 2a) and 4-8 (Figure 2b).

Irrespective of the silicon nitride addition, in all samples only two types of secondary phases were noticed (Figure 2). SEM EDX observations found no boron nitride or any silicon-rich phase in the microstructure. The observed phases match the complex M_2_B borides observed by other researchers investigating steel powders with boron additions [4,6,12,33,34,35]. Two types of borides were noticed. One, with a high concentration of Cr and Fe, is seen as dark agglomerates, and the second, with a high concentration of Cr, Mo, and Fe, is observed as bright “branched” shapes. This observation of chromium, iron, and molybdenum content in borides matches the calculation outcome (Supplementary Data: Figures S20–S23). When Si_3_N_4_ was introduced, some of the borides had facets, suggesting that the grain growth process was modified by the presence of Si_3_N_4_. Borides are the last phase to solidify in the given system; therefore, they occupy places that were left by other phases. This further means that in samples with Si_3_N_4_ at sintering temperature, there was a phase present that mitigated the growth of the austenite grains and resulted in their non-cuboidal shapes.

### 3.2. Density, Porosity, and Dimensional Precision

**Figure 3:**
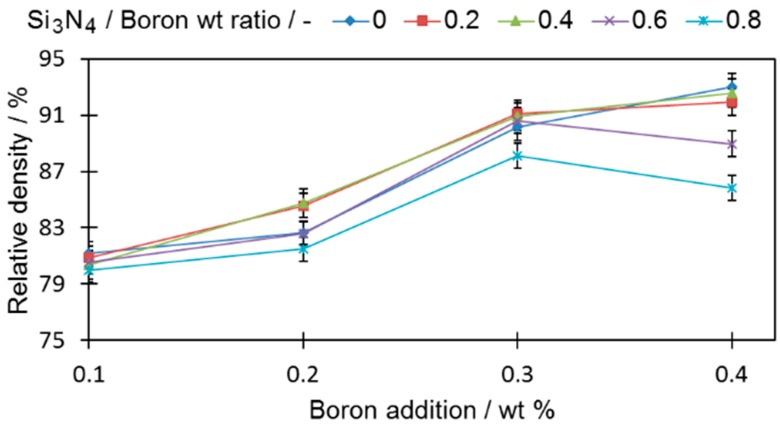
The influence of boron and silicon nitride on the relative density of cylindrical samples. Numerical data are available in Table S2 in Supplementary Material.

In order to evaluate the impact of boron and silicon nitride on the densification properties, cylindrical samples were analyzed (Figure 3).

All tested samples reached a higher relative density than the base sample (0-0) (78.95% ± 0.19%). In all samples with only boron added, if more boron was introduced to the sinters, the density became higher, a fact that is in good agreement with the findings of other researchers [1,6]. For samples with a ratio of silicon nitride to boron greater than 0.4 (0.6 and 0.8), the density decreases, which permits the expectation that the amount of eutectic liquid that acts as a high-speed route for diffusion was reduced. The extent of this drop was greater when a large amount of boron was introduced. It is expected that this is the result of the increased contact probability for those two compounds in the sinter volume during the sintering process. The density decrease is also in agreement with the observation of [24], who showed that Si_3_N_4_, which decomposes at 1127.2 °C, releases free nitrogen that increases porosity in a sinter.

**Figure 4:**
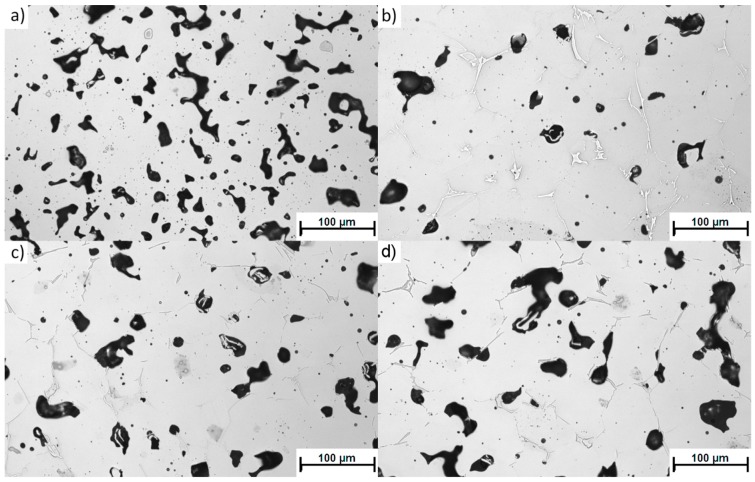
Representative porosity microstructure of samples: (**a**) 0-0; (**b**) 4-0; (**c**) 4-4; and (**d**) 4-8.

**Figure 5:**
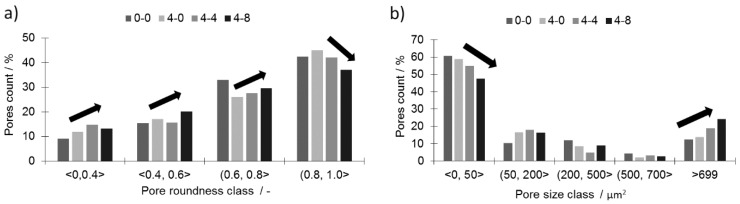
Histograms of the pores: (**a**) roundness; (**b**) area.

The most detrimental sinter microstructure features that affect its properties the most are the porosity and the shape. It is well known that the addition of boron causes a rounding of the pores (sample 4-0, Figure 4b) compared to non-modified 316L (sample 0-0, Figure 4a). This effect was observed in all samples with boron addition—the more boron added, the more pronounced the effect. On the other hand, increasing the silicon nitride addition resulted in an overall increase in porosity (sample 4-4 in Figure 4c, sample 4-8 in Figure 4d) and caused less rounded pore shapes (Figure 5). In this case, the more Si_3_N_4_ added, the more pronounced the effect.

This observation suggests that, as expected, the amount of eutectic liquid under sintering conditions was reduced by the presence of silicon nitride.

All three samples with a 0.4 wt % addition of boron (4-0, 4-4, and 4-8) and an increasing addition of silicon nitride are characterized by a considerably lower porosity than the reference sample (0-0). The histograms of the pore roundness and area are plotted in Figure 5 to estimate the qualitative influence of silicon nitride addition on the sinters. In the tested samples with boron addition (4-0, 4-4, and 4-8), the addition of silicon nitride reduced the fraction of well-rounded pores (0.81 < round < 1.0) while simultaneously increasing the fraction of less-rounded pores (Figure 5a). At the same time, the large pores fraction (>700 µm^2^) increased at the cost of the small pores (<50 µm^2^), as presented in Figure 5b. This means the pores grew larger and became less rounded with the addition of silicon nitride. This may lower the mechanical properties of the given sinters. The decreasing pore roundness also indicates a limited influence of the liquid phase. This limitation may be associated with both a lower overall amount of liquid at the sintering temperature and shortened residence time.

**Figure 6:**
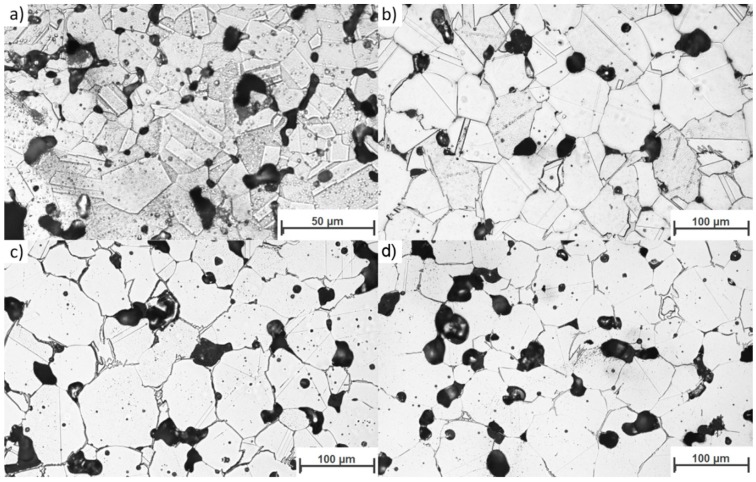
Representative microstructures of selected samples; (**a**) 0-0; (**b**) 4-0; (**c**) 4-4; and (**d**) 4-8.

Microstructural analysis was performed (Figure 6a,d). The grain size analysis showed that even large additions of silicon nitride to the samples modified with boron (sample 4-4 and 4-8) did not alter the average grain diameter (Table 4).

Since large grains grew during liquid phase sintering through dissolution and reprecipitation, one may conclude that the liquid phase was also present when large amounts of silicon nitride were added. Without Si_3_N_4_ powder addition, abnormal grain growth can be seen along with many twins in the large grains. In contrast, average grain growth, no twins in grains, and grain boundary waving can be seen in the samples with Si_3_N_4_ addition. These large grains were interpreted as meaning that the residence time was sufficiently long for the liquid to act and cause the extensive grain growth via the reprecipitation process, which constitutes the final step of the liquid phase influence on the microstructure according to liquid phase theory [8]. Furthermore, this means that a quantity of eutectic liquid was present during the entire sintering cycle, a conclusion that is in good agreement with the observed borides (Figure 2), which show a footprint of the eutectic phase presence.

**Figure 7:**
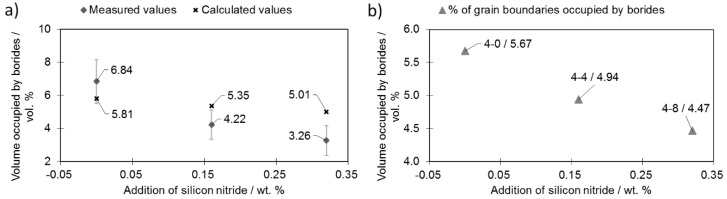
Influence of Si_3_N_4_ addition on the (**a**) amount of borides and (**b**) percentage of grain surface occupied by borides. Data are for selected representative samples with 0.4 wt% boron addition.

Figure 7a presents the total amounts of borides observed in selected samples (containing 0.4 wt% boron) during silicon nitride addition.

Analysis of the microstructure with a focus on the boride distribution confirmed the simulation results (Figure 1)—as more silicon nitride was added, fewer borides were observed (Figure 7a). The measured boride values roughly match the calculated ones. The experimental relationship between the volume occupied by bodies and the Si_3_N_4_ addition deviated from the linear relationship obtained by calculation. This is the effect of the eutectic liquid behavior: When no Si_3_N_4_ is added, the eutectic liquid tends to flow towards the center of the sample [17]. On the other hand, when Si_3_N_4_ is added, flow towards the sample core is impeded and fewer borides are observed in this area and therefore fewer borides are observed in the investigated area.

The data obtained were used to calculate the total fraction of grain boundaries occupied by borides (Figure 7b). This calculation was performed on the assumption that the grains have a regular 20-hedron shape with diameters equal to the mean grain diameter. Based on 100 random boride thickness observations (placed on the grain boundaries), the mean thickness was calculated as 16.5 µm. Porosity was subtracted from the sample volume.

Due to the increasing porosity contribution (Figure 3) along the increasing silicon nitride addition curve, the percentage grain boundaries occupied by borides did not decrease significantly when more silicon nitride was introduced (sample 4-8). Nevertheless, the addition of 0.32 wt % of Si_3_N_4_ (sample 4-8) was enough to reduce the percentage of grain boundaries occupied by borides from 5.67% (sample 4-0) down to 4.47% (sample 4-8).

**Figure 8:**
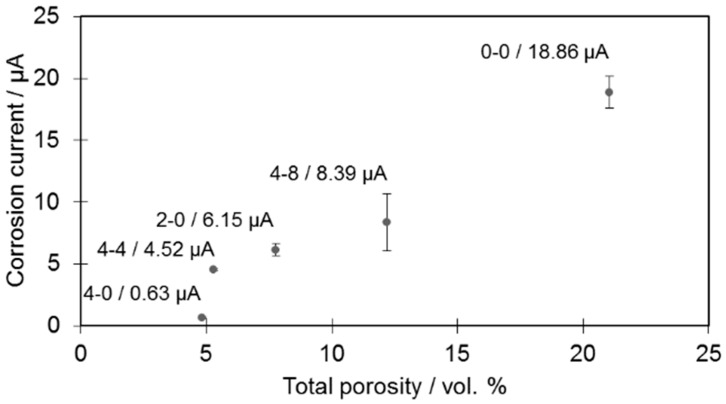
Corrosion current of selected samples as a function of porosity. Numerical data are available in Table S3 in the Supplementary Material.

The microstructural changes caused by additions of boron and silicon nitride (Figure 6) may have a detrimental influence on corrosion resistance behavior (which in this case is one of the most crucial features of the tested material). Corrosion tests were therefore performed only on selected samples (Figure 8).

In the case of the tested samples, it was established that the higher the porosity value, the higher the corrosion current, which is consistent with observations from other researchers [6]. The increase was observed irrespective of the changing amounts of borides present among the samples. This means that the introduced powder modifications do not alter the general characteristics of the material and that pores are mainly responsible for the increased corrosion rate by increasing the contact surface for a corrosive medium.

**Figure 9:**
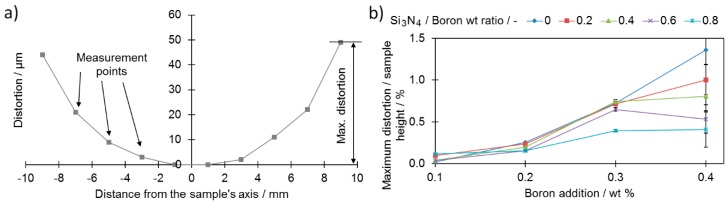
Maximum dimensional distortions of Ø20 × 5 mm cylindrical samples: (**a**) representative example results for sample 4-2; (**b**) as a function of boron and silicon nitride additions. Numerical data are available in Table S4 in the Supplementary Material.

Another feature, which was almost not investigated, is the extent of the dimensional distortions of the sinter—the lower they, are the better. Dimensional distortions are typical for samples with a large boron addition and sintered at a temperature exceeding the eutectic reaction temperature of boron and iron (approximately 1177 °C). This is caused mainly by the eutectic liquid volume, which may lead to separation of neighboring grains by a thin layer of liquid when it exceeds 20% to 25%, and is a consequence of the relative motion of these grains [6,7,35]. The distortions were measured on the pressing side of the samples. A measurement schematic is presented in Figure 9a.

As presented in Figure 9b, all samples, except sample 1-0, were found to present higher maximum dimensional distortions after sintering compared to the base sample (0-0) (0.04% ± 0.002% of the sample height). For samples with a high boron addition (0.4 wt %), the introduction of the silicon nitride resulted in improved dimensional precision (Figure 9b). On the other hand, for samples with a lower addition of boron (0.1 and 0.2 wt %), no significant improvement was noticed along the increased silicon nitride addition curve. This provides information for the case of samples with high boron additions for which the addition of silicon nitride prevented full separation of the neighboring grains responsible for grain reorganization.

When comparing the density values of the cylindrical samples with maximum distortions, a high relative density (Figure 3) may be obtained with relatively low distortions, e.g., sample 4-6.

**Figure 10:**
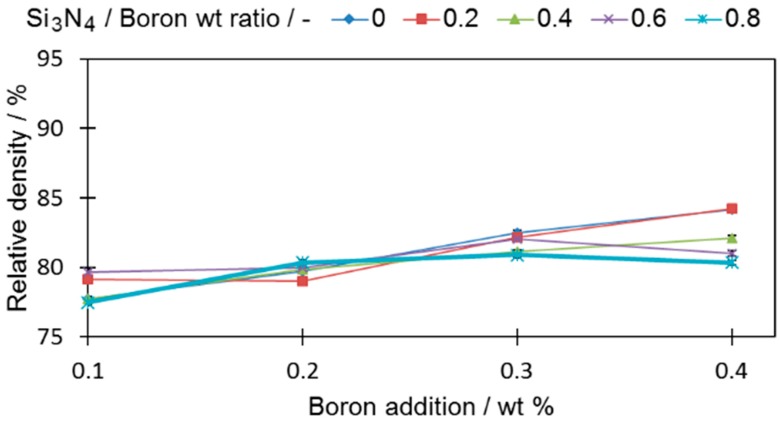
Density change of prismatic samples in the boron and silicon nitride addition functions. Numerical data are available in Table S5 in the Supplementary Material.

In the next step, the TRS samples (30 mm × 12 mm × 6 mm) were tested for density. As presented in Figure 10, noticeably lower densities were obtained for prismatic samples in comparison to cylindrical ones.

This results from the lower compatibility of prismatic samples due to the presence of corners and relatively large outline surfaces compared to the cross-section. Both of these features impede the compaction process and, therefore, decrease the green density. The average green density of the cylindrical sample was 6.3 g*cm^−1^, while the value for the prismatic sample was 5.95 g*cm^−1^ (Table S4 in the Supplementary Data). Despite the altered sample geometry and lower densities, the general sintering mechanisms remained similar. Furthermore, the greater the additions of both boron and Si_3_N_4_, the lower the green density of samples. The same liquid volume was thus not able to fill the whole internal porosity potential and to act with the same efficiency as in cylindrical samples. The density values are presented in relation to fully dense wrought 316L steel.

### 3.3. Mechanical Properties

**Figure 11:**
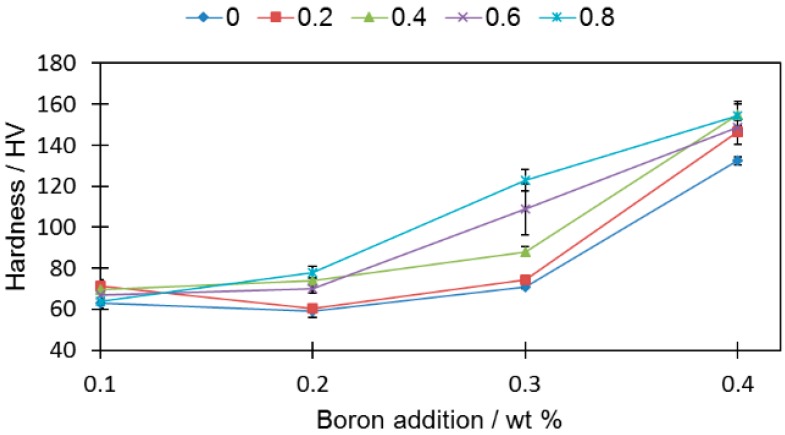
Hardness as a function of boron addition for different silicon nitride additions. Detailed data are available in Table S6 in the Supplementary Data.

Despite having a deteriorating influence on the density (Figure 10), the silicon nitride addition resulted in increased hardness (Figure 11). In general, all the tested samples (aside from samples 2-0 and 2-2) were characterized with a higher hardness than the reference sample (0-0) (HV 60.8 ± 1.2).

For the rest of the samples, the hardness increase was independent of the boron addition. It was also irrespective of the porosity, i.e., hardness grows even the when porosity drops. Sample 4-8 had an over two times higher (HV 154 ± 6.9) hardness value than sample 0-0 (HV 60.8 ± 1.2), despite comparable relative densities (80.35% ± 0.18% and 79.62% ± 0.19%, respectively).

The combined influence of the porosity change and the reduced number of secondary phases, together with the varying additions of boron and silicon nitride, were checked by performing standardized transverse rupture strength (TRS) tests.

**Figure 12:**
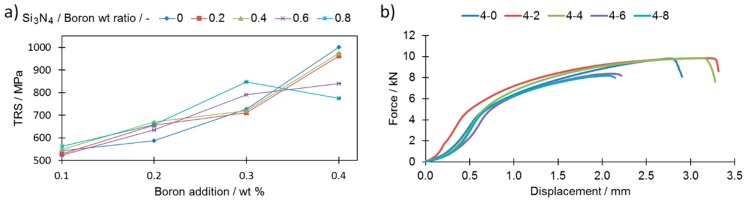
(**a**) Influence of Si_3_N_4_ and boron additions on transverse rupture strength (TRS) (**b**) representative bending curves of selected samples. Numerical data are available in Table S7 in the Supplementary Material.

As a general rule, the stronger the interconnections among neighboring grains, the higher the TRS. Although the highest TRS value was registered for sample 4-0, this was severely distorted; this powder composition is thus considered to be technologically useless. By comparing samples from extreme cases, i.e., 0-0 and 4-8, which had roughly similar relative densities (79.62 and 80.34), respectively, one can observe a significant increase in the TRS value, from 457 MPa for sample 0-0 to 775 MPa for sample 4-8 (Figure 12a,b). As presented in Figure 12a, the addition of silicon nitride to the boron-modified samples increases the TRS value only when the boron addition does not exceed 0.2 wt% and if the Si_3_N_4_ to boron ratio is below 0.4. When higher amounts of boron and/or Si_3_N_4_ are added, then this effect is not visible anymore. For higher Si_3_N_4_ additions (Si_3_N_4_ to boron ratio = 0.6), the TRS value is always higher than for samples without Si_3_N_4_. When the ratio reaches 0.8, one may observe a drastic drop of the TRS. The same effect was observed in terms of displacement during the TRS test (Figure 9b).

A change of this kind was possible due to a percentage drop in the grain boundaries occupied by borides as presented in Figure 7b. This increase was achieved despite an unfavorable decrease in the pore roundness value and an increase in their volume together with an increasing Si_3_N_4_ addition (Figure 5a,b).

### 3.4. Summary

**Figure 13:**
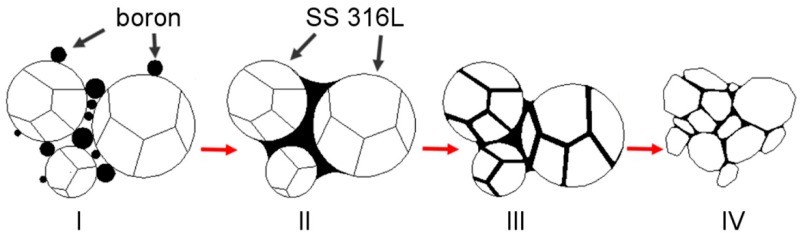
Microstructural phenomena occurring during the sintering process of boron-doped samples (without silicon nitride addition). **I**: Mixed state; **II**: Eutectic liquid creation and primary reorganization; **III**: Penetration of the grain boundaries; **IV:** Secondary reorganization.

**Figure 14:**
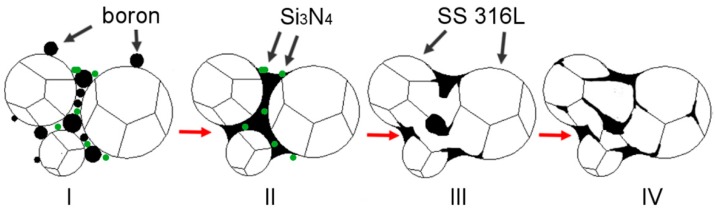
Proposed explanation for microstructural phenomena occurring during the sintering process of boron-doped samples with of silicon nitride addition. **I**: Mixed state; **II**: Eutectic liquid creation and primary reorganization; **III**: Reaction between silicon nitride and boron resulting in the creation of enhanced interconnections among the particles; **IV**: Penetration of the grain boundaries with retained secondary reorganization.

A general explanation is proposed and described in detail in a subsequent part of this work to explain the observed influence of silicon nitride addition on samples modified with boron addition. When boron is added to the ferrous metal powder and heated up, a eutectic reaction takes place. As a result, a eutectic liquid phase is created and, due to the insolubility of boron in the matrix, the liquid persists as long as the temperature is higher than its solidus temperature. A generally accepted microstructural impact of a persistent liquid phase on green compact, which was described in Figure 13 [6,7,35], is, in this case, altered by the presence of silicon nitride (Figure 14).

The presence of the eutectic liquid at the sintering temperature depends to a significant extent on the boron concentration along the liquidus lines surrounding the eutectic point. When a nitrogen atom combines with a boron atom from E(M_2_B + M) (where M = Fe, Cr, Mo), an in-situ h-BN molecule is created, leading to the expulsion of three selected metal atoms: Cr, Fe, and/or Mo [2]. All of these also solidify on the neighboring grains due to the lack of boron and create mutual interconnections without borides.

This effect may be seen in Figure 7b, where the total percentage of grain boundaries occupied by borides drops from 5.67% (sample 4-0) to 4.47% (sample 4-8). This means that enhanced interconnections among grains were created on over 1.2% of all grain boundaries (a relative increase of 21%). These interconnections create solid joints among the grains, preventing them from reorganization in the initial sintering step, which in turn decreases distortions during sintering. This mechanism was present in all tested samples to various extents depending on the amounts of added boron and Si_3_N_4_.

Equation (2) describes a general mechanism of boride formation in AISI 316L steel modified with boron; this is in good agreement with the microstructural observations (Figure 7a,b). Based on the agreement between the decreasing borides amounts and the amount calculations presented in Figure 7a,b, one may conclude that Equation (3) is no longer valid when silicon nitride is added. An interaction between the eutectic liquid and the Si_3_N_4_ occurs as proposed in Equation (3):

(2) $$B+\gamma \;\vec{T\;incr.}\;\gamma +\;E\left(M_{2}B+M\right)\;\vec{\;T\;decr.}\;\;\gamma \;+M_{2}B\;TETR\;+\;Cr_{2}B\;ORTH,$$

(3) $$B+\gamma +Si_{3}N_{4\;\;}\vec{T\;incr.}\;\;E\left(M_{2}B+M\right)+\;Si_{3}N_{4\;\;}\vec{\;T\;incr.}\;\gamma \;+\;E\left(M_{2}B+M\right)+\;BN\;\;\vec{\;T\;decr.\;\;}\;\gamma \;+M_{2}B\;TETR\;+\;Cr_{2}B\;ORTH+\;BN,$$

where *M* is Fe, Cr, or Mo and *T* is temperature (°C).

Opposite observations were presented in Grądzka-Dahlke et al. (2007) [36], where Co-Cr-Mo powder was modified with B_4_C and Si_3_N_4_ additions were added independently. Neither of these compounds resulted in an increased sinter density during the sintering process. This result was due to the high thermodynamic stability of B_4_C, due to which the boron could not interact with the matrix and, as a result, no eutectic reaction occurred. In the case of a separate Si_3_N_4_ addition, the density was also not increased due to lack of chemical interaction with the matrix. In both cases, large interconnected pores were spotted in the microstructure, which hindered the diffusion process.

## 4. Conclusions

It is reported here that a reduction of borides originating from the eutectic reaction is possible by the introduction of silicon nitride. It was shown that by combining additions of boron and Si_3_N_4_ in various proportions to AISI 316L stainless steel, one may control a sinter’s distortions and its transverse rupture strength by direct control of the amount of solidified secondary phases (originating from the eutectic liquid phase). The interaction between boron and silicon nitride was observed globally as the boride amount lowered and its morphology changed. It was not, however, successful in providing direct observations of the products of this reaction (i.e., BN). The greater the silicon nitride addition to a sinter modified with boron addition, the lower the maximum distortions were that followed from the sintering process. The TRS value was successfully increased from 457 MPa for a base sample to 775 MPa for sample 4-8 while keeping the maximum distortions at a relatively low level (200 µm). An increase in the mechanical properties was assigned to the combined effect of the formation of enhanced interconnections among the grains and the presence of borides.

## Figures and Tables

**Table 1:** Chemical composition of AISI 316L.

Element	C	Cr	Ni	Mo	Si	Mn	O *	N	Fe
Concentration [wt%]	0.02	16.8	13.0	2.2	0.85	0.1	0.2	0.04	Balance

* oxygen is mainly present in the form of oxides on particle surfaces.

**Table 2:** Grain size distribution of AISI 316L powder.

Grain size class [μm]	−45	+45	+53	+75	+106	+125	+150
Frequency [%]	40.7	10.8	21.1	17.2	5.5	3.8	0.9

**Table 3:** Description of samples. For the convenience of the reader, the first digit describes the amount of boron added in tenths of a percent while the second digit describes the weight relation of Si_3_N_4_ to boron, i.e., 3–6 means: 0.3 wt % of boron and the weight of Si_3_N_4_ is equal to 0.18% (0.6 · 0.3 wt %).

		Boron Addition [wt %]				
		0.0	0.1	0.2	0.3	0.4
Si_3_N_4_ to Boron weight ratio	0.0	0-0	1–0	2–0	3–0	4–0
	0.2	-	1–2	2–2	3–2	4–2
	0.4	-	1–4	2–4	3–4	4–4
	0.6	-	1–6	2–6	3–6	4–6
	0.8	-	1–8	2–8	3–8	4–8

**Table 4:** Comparison of average grain sizes for selected samples.

Sample code	0	4-0	4-4	4-8
average grain size [µm]	18.10 ± 0.21	65.92 ± 0.56	66.44 ± 0.79	66.58 ± 0.62

## Supplementary Materials

The following are available online at https://www.mdpi.com/1996-1944/12/11/1798/s1, Figure S1: Scheil–Gulliver solidification plot of sample 0-0, Figure S2: Scheil–Gulliver solidification plot of sample 1-0, Figure S3: Scheil–Gulliver solidification plot of sample 1-2, Figure S4: Scheil–Gulliver solidification plot of sample 1-4, Figure S5: Scheil–Gulliver solidification plot of sample 1-6, Figure S6: Scheil–Gulliver solidification plot of sample 1-8, Figure S7: Scheil–Gulliver solidification plot of sample 2-0, Figure S8: Scheil–Gulliver solidification plot of sample 2-2, Figure S9: Scheil–Gulliver solidification plot of sample 2-4, Figure S10: Scheil–Gulliver solidification plot of sample 2-6, Figure S11: Scheil–Gulliver solidification plot of sample 2-8, Figure S12: Scheil–Gulliver solidification plot of sample 3-0, Figure S13: Scheil–Gulliver solidification plot of sample 3-2, Figure S14: Scheil–Gulliver solidification plot of sample 3-4, Figure S15: Scheil–Gulliver solidification plot of sample 3-6, Figure S16: Scheil–Gulliver solidification plot of sample 3-8, Figure S17: Scheil–Gulliver solidification plot of sample 4-2, Figure S18: Scheil–Gulliver solidification plot of sample 4-4, Figure S19: Scheil–Gulliver solidification plot of sample 4-6, Figure S20: The composition of Cr2B in sample 4-0, Figure S21: The composition of Cr2B in sample 4-8, Figure S22: The composition of M2B in sample 4-0, Figure S23: The composition of M2B in sample 4-8, Table S1: The calculations of secondary phases amount depending on the chemical composition of the samples, Table S2: The influence of boron and silicon nitride on the relative density of cylindrical samples, Table S3: Corrosion current of selected samples as a function of porosity, Table S4: Maximum dimensional distortions of Ø20 × 5 mm cylindrical samples as a function of boron and silicon nitride additions, Table S5: Density change of prismatic samples in the boron and silicon nitride addition functions, Table S6: Hardness as a function of boron addition for different silicon nitride additions, Table S7: Influence of Si3N4 and boron additions on transverse rupture strength (TRS).
