# Supplementary material for: Improving the Dimensional Stability and Mechanical Properties of AISI 316L + B Sinters by Si3N4 Addition
Source: Materials (Basel). 2019 Jun 3;12(11):1798. doi: 10.3390/ma12111798 (PMC6600792; doi:10.3390/ma12111798)
Supplement: Supplementary file 1 [file materials-12-01798-s001.pdf]

materials

Supplementary Information

Improving the Dimensional Stability and Mechanical Properties of AISI 316L + B Sinters by Si_3N_4 Addition

Mateusz Skałoń[1,2], Ricardo Buzolin[1], Jan Kazior[2], Christof Sommitsch[1] and Marek Hebda[2*]

1 IMAT Institute of Materials Science, Joining and Forming, Graz University of Technology, Kopernikusgasse 24/1, 8010 Graz, Austria; mateusz.skalon@tugraz.at (M.S.); ricardo.buzolin@tugraz.at (R.B.); christof.sommitsch@tugraz.at (C.S.)
2 Institute of Materials Engineering, Cracow University of Technology, Cracow, 24 Warszawska ave, 31-155, Poland; kazior@mech.pk.edu.pl (J.K.)
* Correspondence: mhebda@pk.edu.pl (M.H.); Tel.: +48 126283423

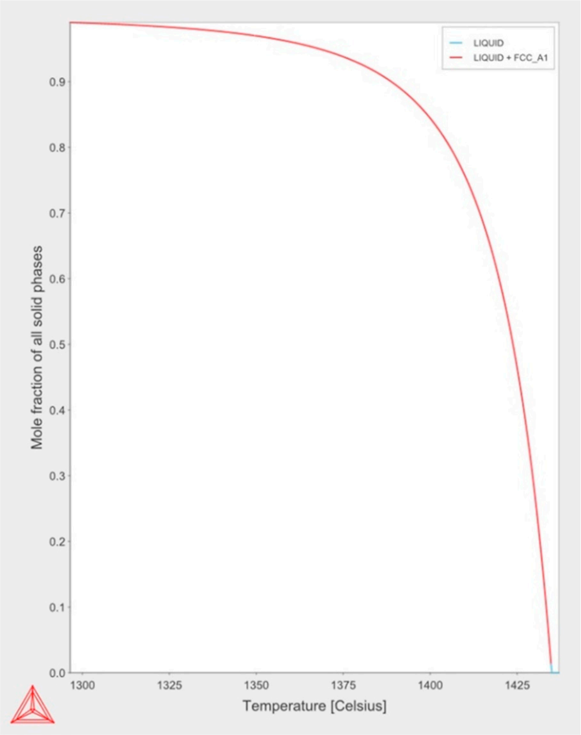

Figure S1. Scheil–Gulliver solidification plot of sample 0-0.

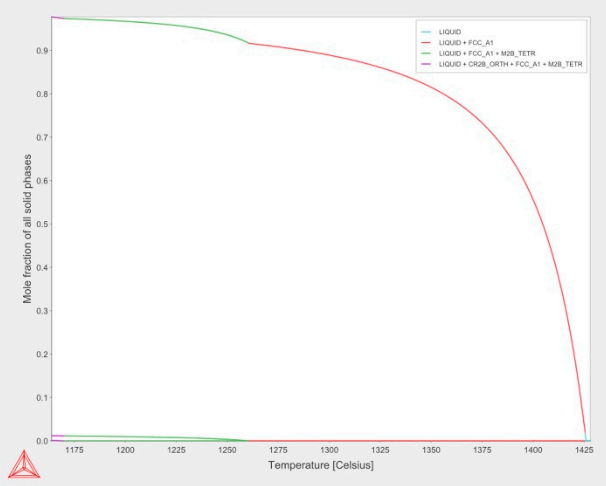

Figure S2. Scheil–Gulliver solidification plot of sample 1-0.

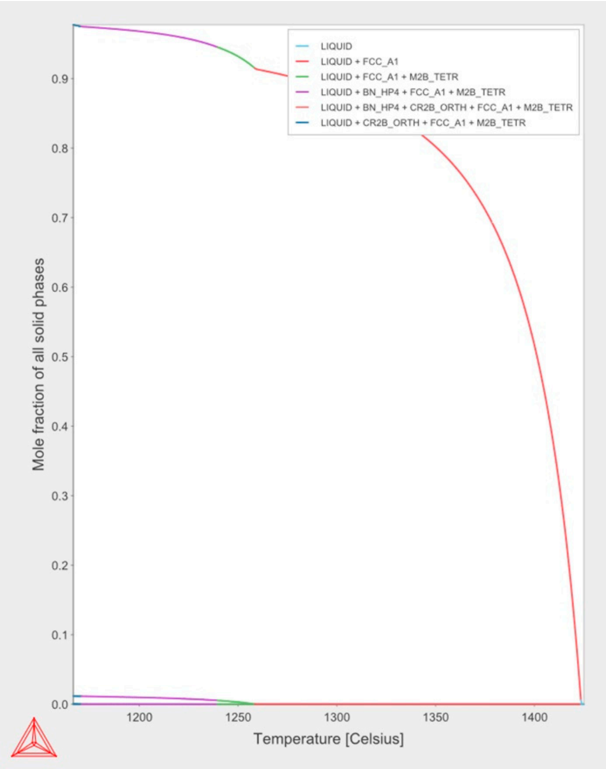

Figure S3. Scheil–Gulliver solidification plot of sample 1-2.

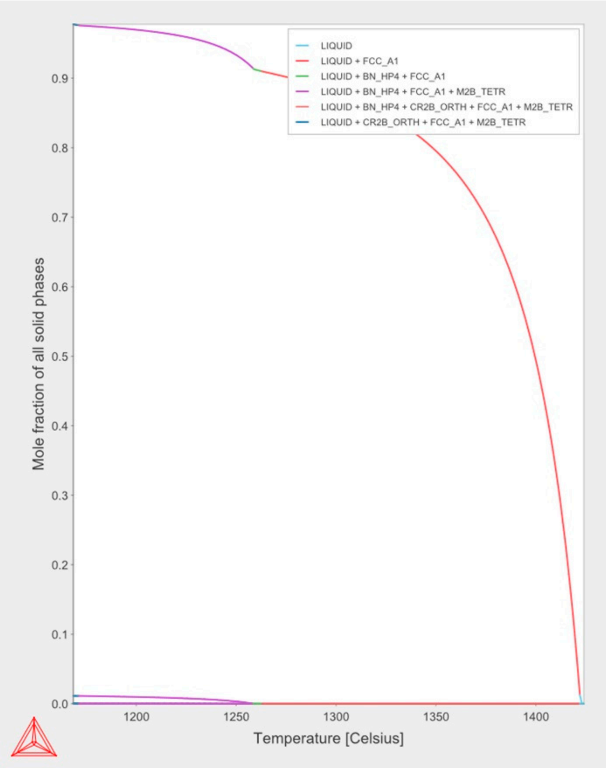

Figure S4. Scheil–Gulliver solidification plot of sample 1-4.

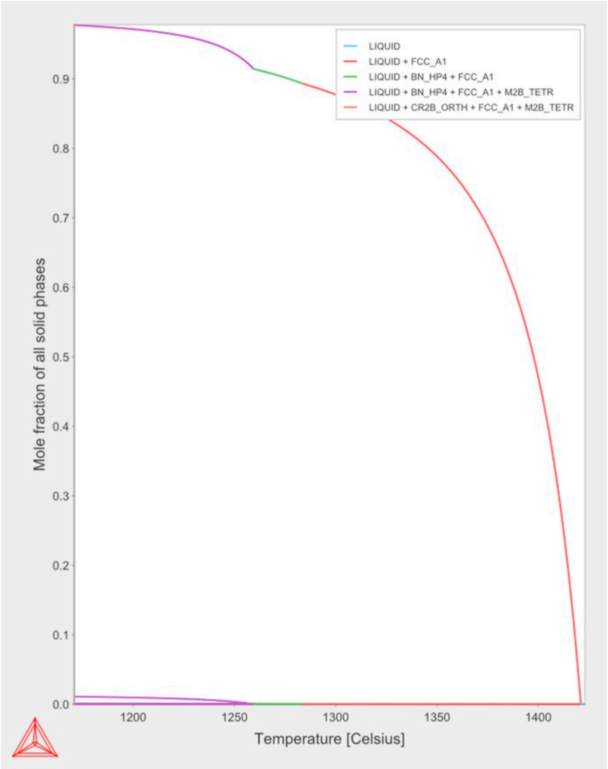

Figure S5. Scheil–Gulliver solidification plot of sample 1-6.

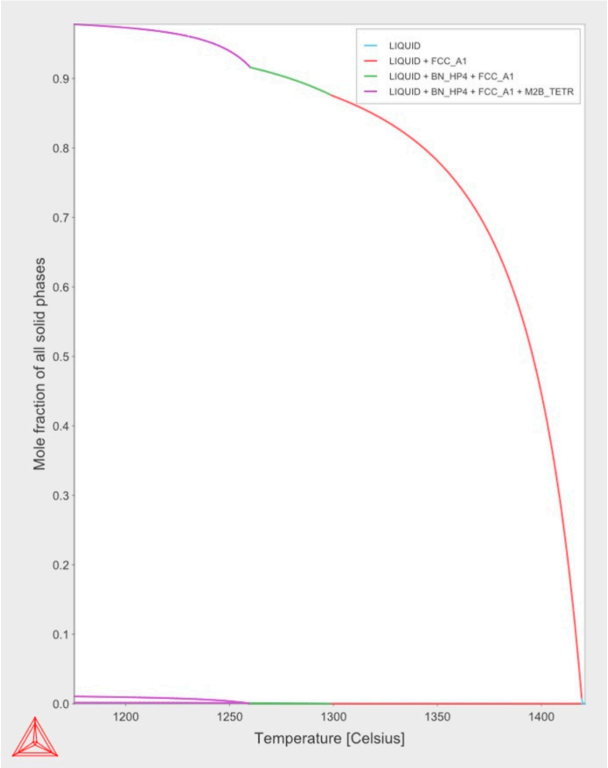

Figure S6. Scheil–Gulliver solidification plot of sample 1-8.

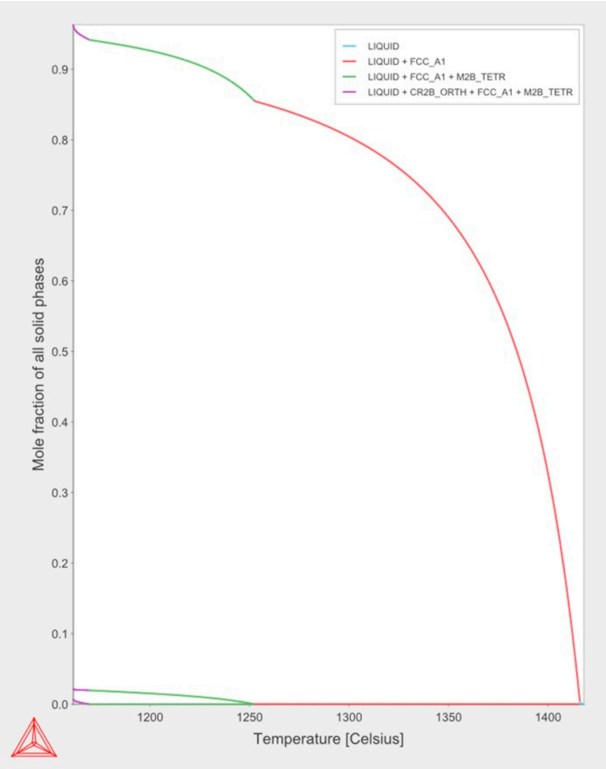

Figure S7. Scheil–Gulliver solidification plot of sample 2-0.

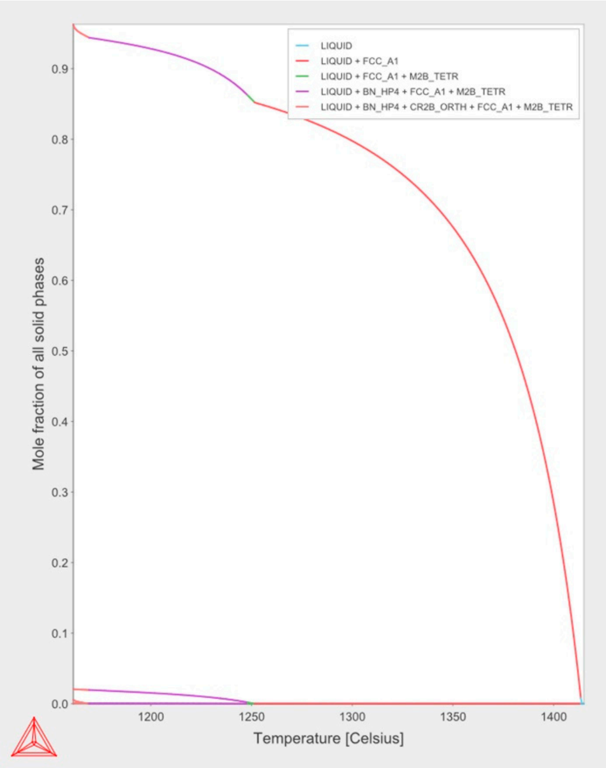

Figure S8. Scheil–Gulliver solidification plot of sample 2-2.

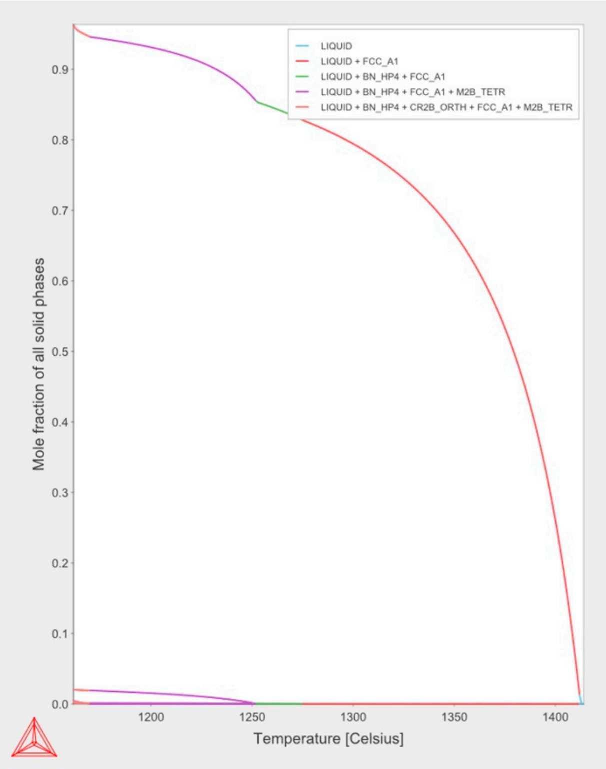

Figure S9. Scheil–Gulliver solidification plot of sample 2-4.

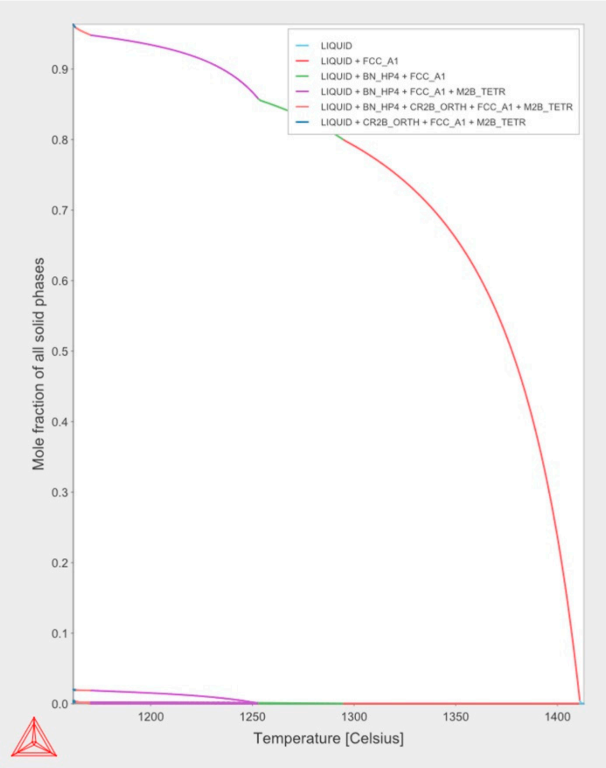

Figure S10. Scheil–Gulliver solidification plot of sample 2-6.

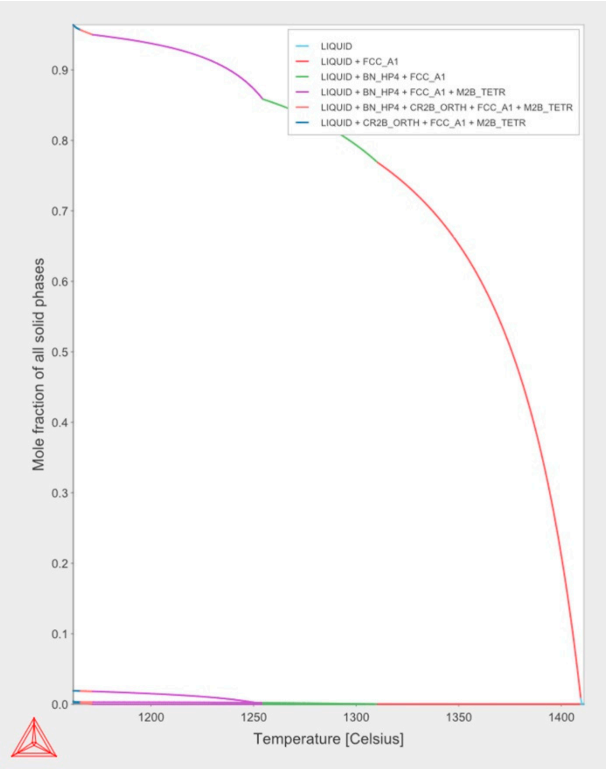

Figure S11. Scheil–Gulliver solidification plot of sample 2-8.

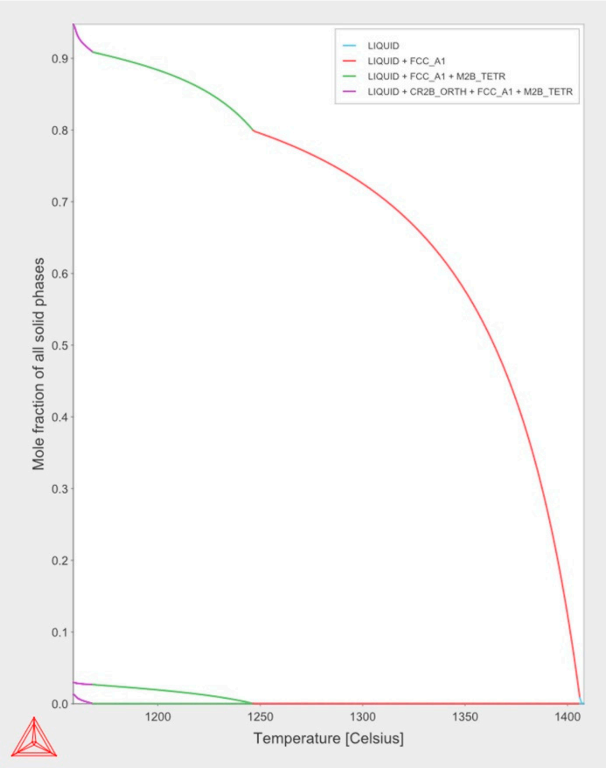

Figure S12. Scheil–Gulliver solidification plot of sample 3-0.

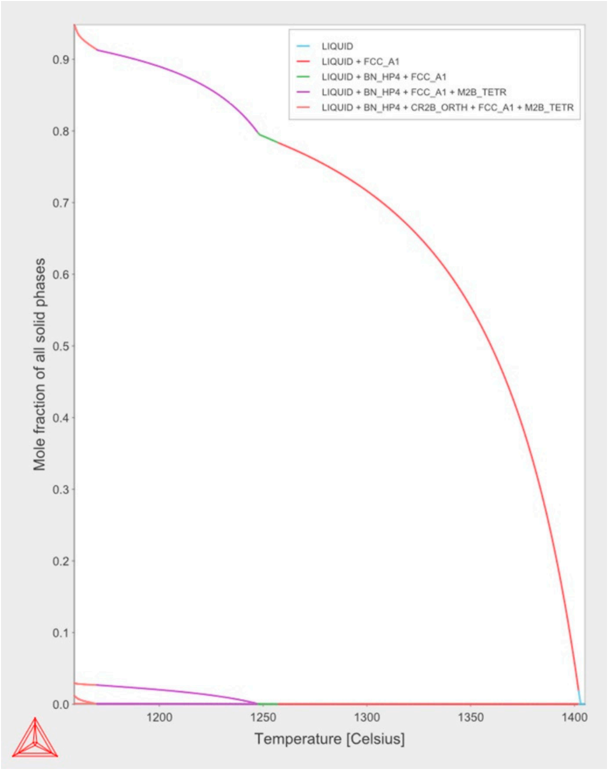

Figure S13. Scheil–Gulliver solidification plot of sample 3-2.

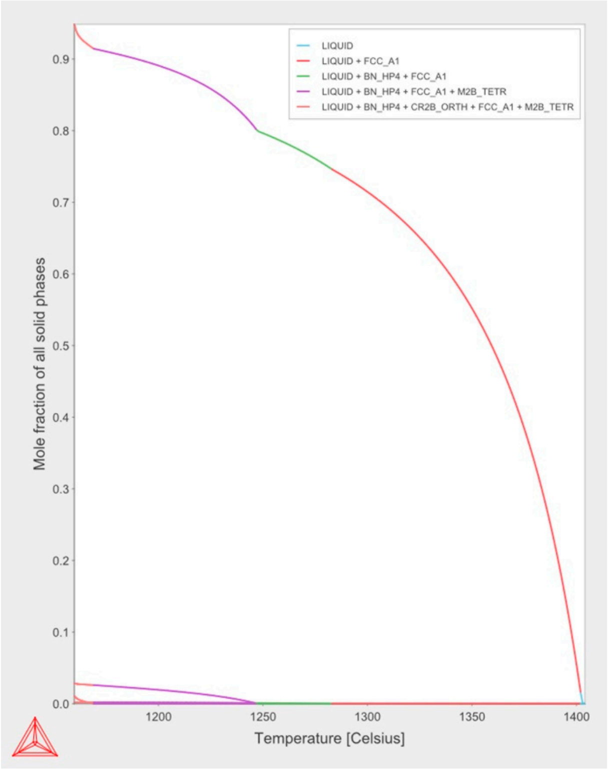

Figure S14. Scheil–Gulliver solidification plot of sample 3-4.

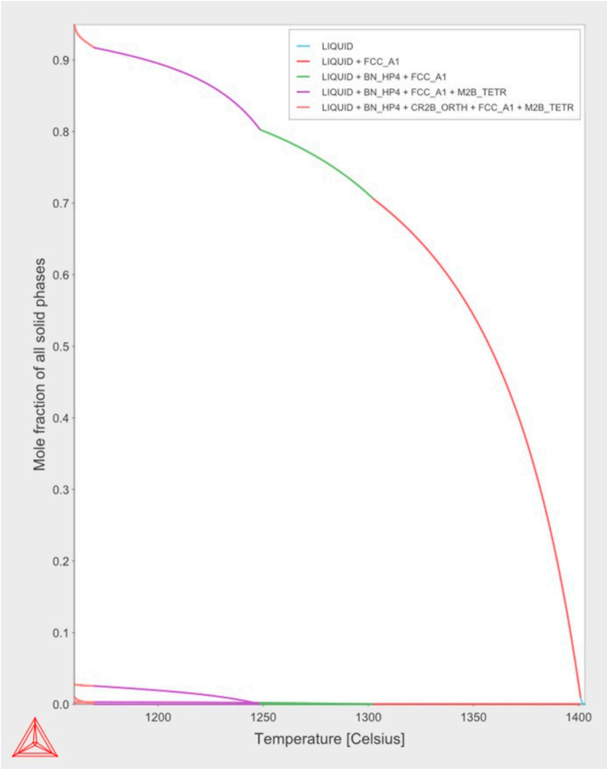

Figure S15. Scheil–Gulliver solidification plot of sample 3-6.

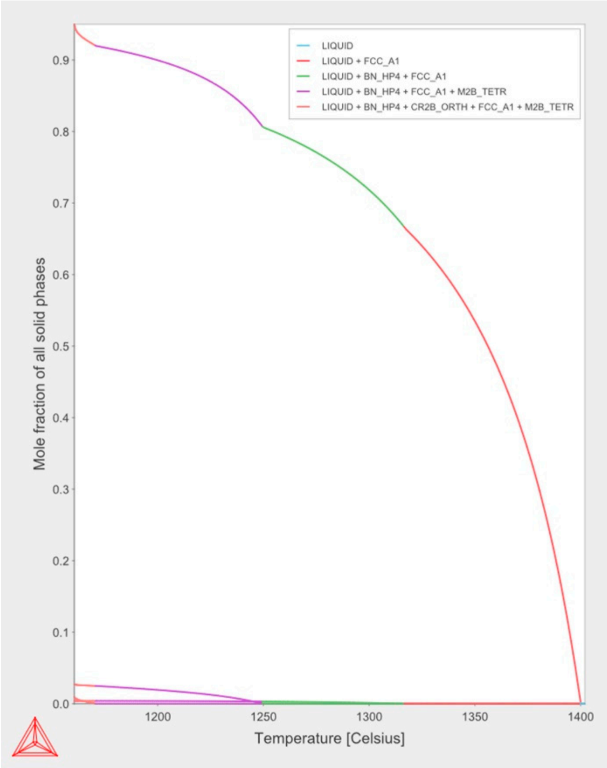

Figure S16. Scheil–Gulliver solidification plot of sample 3-8.

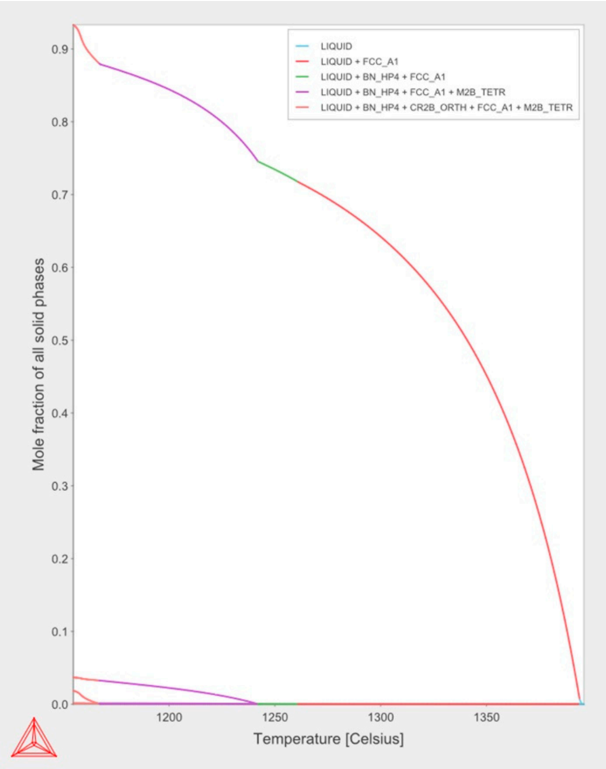

Figure S17. Scheil–Gulliver solidification plot of sample 4-2.

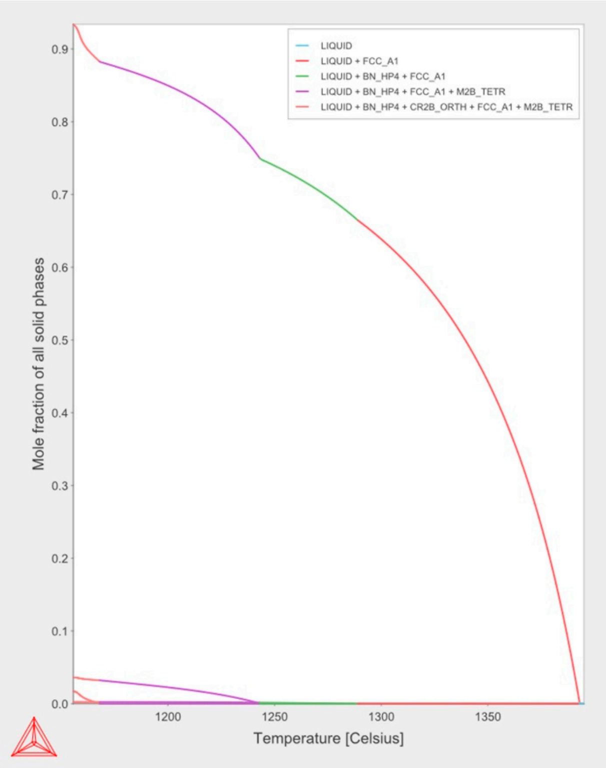

Figure S18. Scheil–Gulliver solidification plot of sample 4-4.

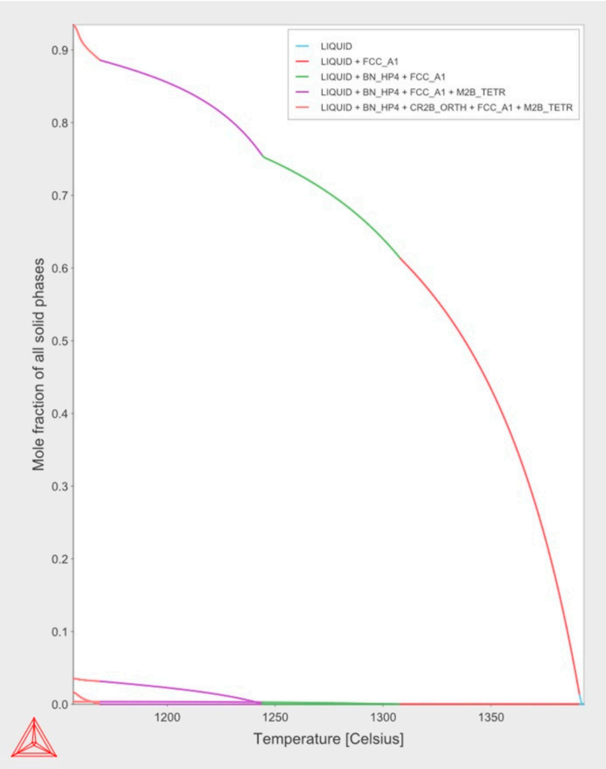

Figure S19. Scheil–Gulliver solidification plot of sample 4-6.

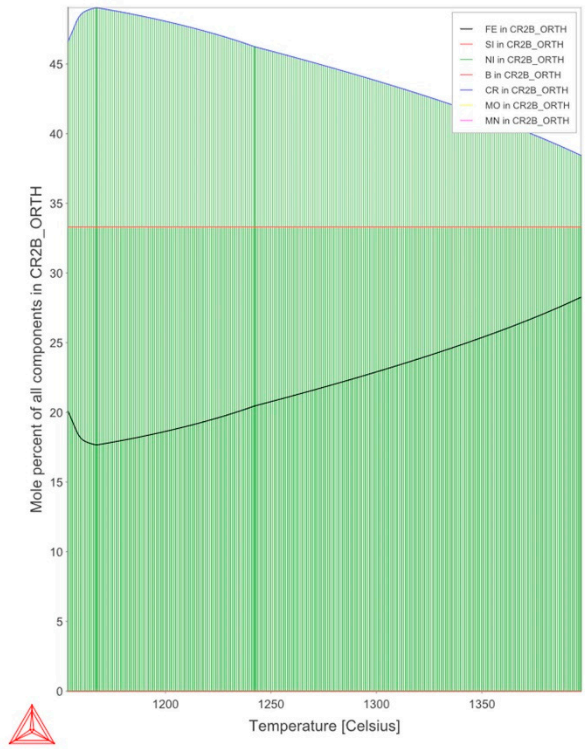

Figure S20. The composition of Cr_2B in sample 4-0.

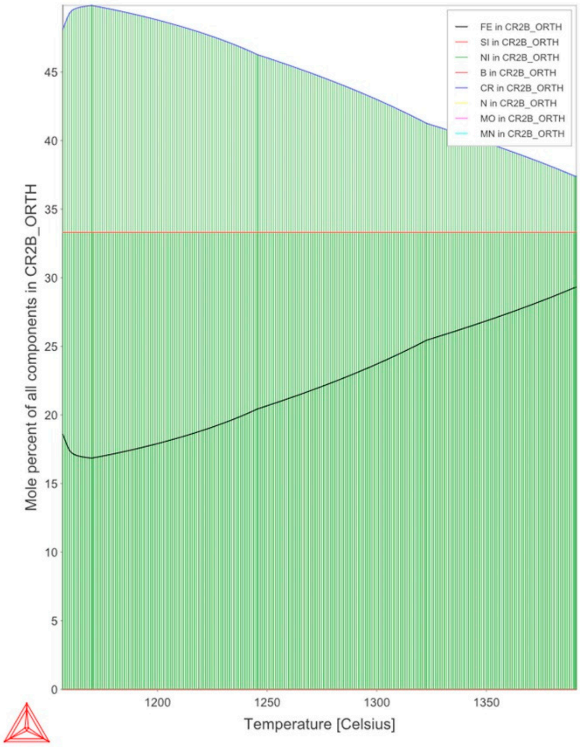

Figure S21. The composition of Cr_2B in sample 4-8.

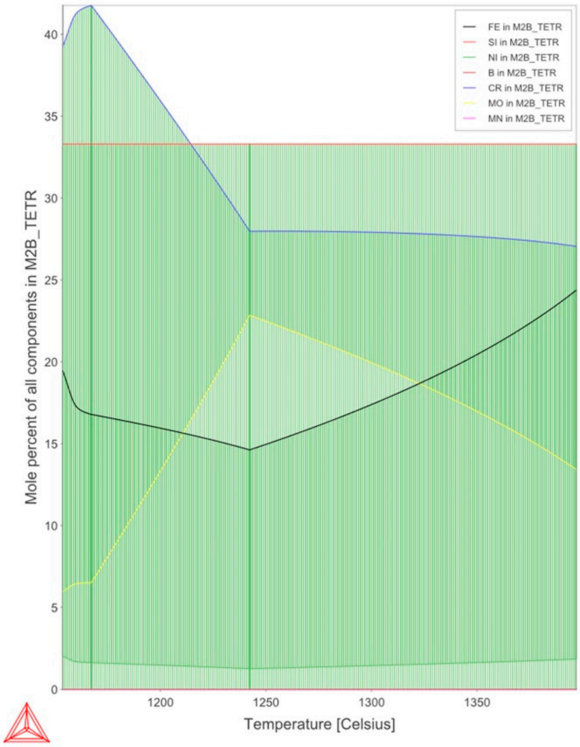

Figure S22. The composition of M_2B in sample 4-0.

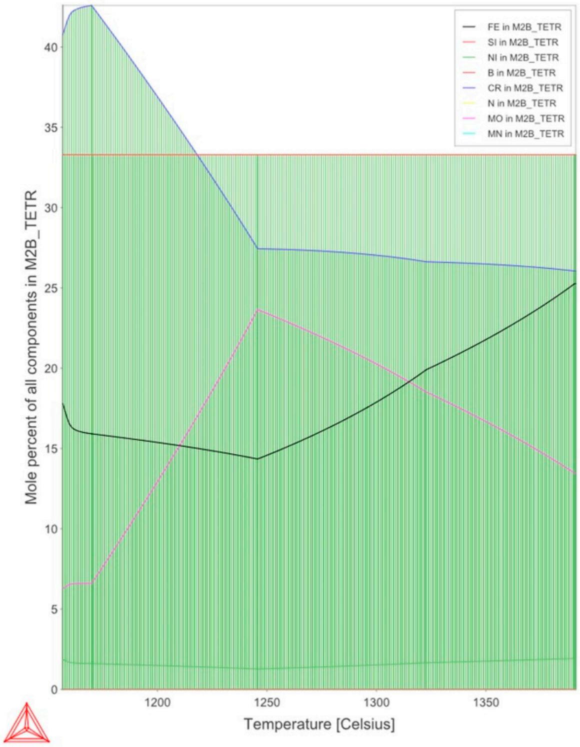

Figure S23. The composition of M_2B in sample 4-8.

Table S1. The calculations of secondary phases amount depending on the chemical composition of the samples.

		Description of samples																				
		0-0	1-0	1-2	1-4	1-6	1-8	2-0	2-2	2-4	2-6	2-8	3-0	3-2	3-4	3-6	3-8	4-0	4-2	4-4	4-6	4-8
Phase amount / mole %	M_2B	0.000	1.166	1.168	1.152	1.101	0.986	2.126	2.068	2.058	1.994	1.928	2.953	2.965	2.852	2.790	2.727	3.783	3.720	3.614	3.540	3.452
	Cr_2B	0.000	0.117	0.075	0.062	0.026	0.000	0.704	0.589	0.543	0.474	0.434	1.322	1.181	1.086	1.045	1.017	2.024	1.879	1.737	1.631	1.556
	BN	0.000	0.000	0.025	0.074	0.104	0.077	0.000	0.075	0.126	0.212	0.307	0.000	0.126	0.185	0.280	0.411	0.000	0.160	0.255	0.379	0.490

Table S2. The influence of boron and silicon nitride on the relative density of cylindrical samples.

		Boron addition / wt%				
		0.0	0.1	0.2	0.3	0.4
Si_3N_4/B mass ratio / -	0.0	78.95±0.19	81.17±0.22	82.63±0.22	90.14±0.25	93.01±0.26
	0.2	-	80.90±0.22	84.56±0.23	91.13±0.25	91.93±0.26
	0.4	-	80.36±0.21	84.75±0.23	90.90±0.25	92.57±0.26
	0.6	-	80.56±0.21	82.60±0.23	90.61±0.25	88.97±0.24
	0.8	-	79.96±0.21	81.47±0.22	88.10±0.24	85.83±0.23

Table S3. Corrosion current of selected samples as a function of porosity.

Description of samples	I_{corr}	I_{corr} st.dev.	E_{corr}	E_{corr} st.dev.
	μA		mV	
0-0	18.86	1.271	-305.2	1.144
2-0	6.15	0.475	-315.8	0.330
4-0	0.63	0.066	-272.4	8.674
4-4	4.52	0.051	-338.7	7.270
4-8	8.39	4.624	-320.7	5.564

Table S4. Maximum dimensional distortions of Ø20 × 5 mm cylindrical samples as a function of boron and silicon nitride additions.

		Boron addition / wt %				
		0.0	0.1	0.2	0.3	0.4
Si_3N_4/B mass ratio / -	0.0	20±10	10±10	130±10	360±10	650±10
	0.2	-	50±10	120±10	350±10	490±10
	0.4	-	20±10	100±10	370±10	380±10
	0.6	-	20±10	80±0.01	320±10	260±10
	0.8	-	60±10	80±10	200±10	200±10

Table S5. Density change of prismatic samples in the boron and silicon nitride addition functions.

		Boron addition / wt %				
		0.0	0.1	0.2	0.3	0.4
Si_3N_4/B molar ratio / -	0.0	79.62±0.19	77.71± 0.18	79.72±0.19	82.52±0.18	84.15±0.20
	0.2	-	79.14±0.18	79.04±0.19	82.16±0.18	84.25±0.19
	0.4	-	77.76±0.19	79.86±0.18	81.12±0.19	82.12±0.19
	0.6	-	79.65±0.19	79.97±0.18	82.05±0.19	81.02±0.19
	0.8	-	77.48±0.18	80.35±0.19	80.91±0.19	80.35±0.18

Table S6. Hardness as a function of boron addition for different silicon nitride additions.

		Boron addition / wt %			
Si_3N_4 addition / wt %	Si_3N_4/B wt ratio / -	0.1	0.2	0.3	0.4
0	0	62.9 ± 2.4	58.9 ± 3.0	71.1 ± 0.9	132.3 ± 2.1
0.08	0.2	71.4 ± 3.0	60.5 ± 1.4	74.5 ± 2.0	146.3 ± 6.0
0.16	0.4	69.6 ± 4.4	74.0 ± 1.3	88.0 ± 2.4	154.7 ± 5.2
0.24	0.6	66.9 ± 5.1	69.9 ± 2.0	108.7 ± 12.4	148.7 ± 0.9
0.32	0.8	64.0 ± 0.7	77.9 ± 2.9	123.0 ± 5.4	154.3 ± 6.9

Table S7. Influence of Si_3N_4 and boron additions on transverse rupture strength (TRS).

		Boron addition / wt %				
		0.0	0.1	0.2	0.3	0.4
Si_3N_4/B mass ratio / -	0.0	457±9	544±11	587±13	728±12	1002±23
	0.2	-	529±11	656±9	711±11	963±35
	0.4	-	548±7	670±17	720±18	975±26
	0.6	-	524±15	634±11	791±6	841±13
	0.8	-	563±15	658±14	847±15	775±5
